# A review of Aconiti Lateralis Radix Praeparata (Fuzi) for kidney disease: phytochemistry, toxicology, herbal processing, and pharmacology

**DOI:** 10.3389/fphar.2024.1427333

**Published:** 2024-07-03

**Authors:** Ziyang Wu, Jiawen Qian, Chenhang Feng, Zhouqi Chen, Xiangfu Gao, Yang Liu, Yuancheng Gao

**Affiliations:** ^1^ Department of Nephrology, The First Affiliated Hospital of Zhejiang Chinese Medical University (Zhejiang Provincial Hospital of Chinese Medicine), Hangzhou, China; ^2^ The Third Affiliated Clinical Medical College, Zhejiang Chinese Medical University, Hangzhou, China; ^3^ Shaanxi Academy of Traditional Chinese Medicine, Xi’an, China

**Keywords:** Aconiti Lateralis Radix Praeparata (Fuzi), processing, kidney disease, pharmacology, toxicology, phytochemistry

## Abstract

**Background:**

Aconiti Lateralis Radix Praeparata, commonly known as Fuzi in. traditional Chinese medicine (TCM), is widely utilized in clinical practice despite its inherent toxicity. Since ancient times, TCM practitioners have explored various processing techniques to broaden its clinical applications and enhance its safety profile. This review aims to summarize the effects of processing on the chemical composition, toxicity, and pharmacological properties of Fuzi, as well as investigate potential underlying mechanisms.

**Methods:**

Data on phytochemistry, toxicology, pharmacology, and processing methods of Fuzi were gathered from the literature of electronic databases, including Web of Science, PubMed, and CNKI.

**Results:**

Fuzi contains over 100 kinds of chemical compounds, including alkaloids, flavonoids, and polysaccharides, among which alkaloids are the main active compounds. Diester-diterpenoid alkaloids are the main contributors to Fuzi’s toxicity and have side effects on some organs, such as the heart, liver, kidneys, nervous system, and reproductive system. The chemical composition of aconite, particularly its alkaloid content, was changed by hydrolysis or substitution reaction during processing to enhance its efficacy and reduce its toxicity. Salted aconite could enhance the therapeutic efficacy of Fuzi in treating kidney diseases and influence its pharmacokinetics.

**Conclusion:**

Processing plays an important role in increasing the efficiency and decreasing toxicity of aconite. Further studies are needed to elucidate the changes of aconite before and after processing and the underlying mechanisms of these changes, thereby providing evidence for the clinical safety of drug use.

## 1 Introduction

Aconiti Lateralis Radix Praeparata, the lateral root of *Aconitum carmichaelii* Debeaux, is a traditional Chinese medicine (TCM), renowned for its significant bioactivities and high toxicity. This herb, known as “Fuzi” in Chinese, “Bushi” in Japanese, and “Kyeong-Po Buja” in Korean, is prevalent across many Asian countries and is frequently cited in traditional medical classics (Kondo et al., 2022; Wu et al., 2022).

Historically, as detailed in Shennong’s *Classic of Materia Medica* from the Han Dynasty, Fuzi was used for its warming property and pungent taste. It was prescribed for wind-cold dispersal, cough with dyspnea from pathogenic qi, warming the spleen and lungs, healing trauma, resolving abdominal masses and circulatory disorders, and alleviating limb spasms and pain caused by cold-damp conditions (Li et al., 2023; Yuan and Yuan, 2023).

Recent pharmacological studies have explored Fuzi’s active compounds, which have shown potential in anti-inflammatory and anti-tumor activities, cardiovascular and renal protection, and immune enhancement.

Despite its therapeutic benefits, the pronounced toxicity of Fuzi limits its clinical application compared to other TCM herbs. Therefore, optimizing the balance between efficacy and toxicity through various processing methods has emerged as a critical area of research. This review examines the different processing techniques applied to Fuzi, focusing on how these methods enhance its pharmacological effects and reduce its toxicity, such as salt processing to improve renal benefits. Moreover, it discusses the outcomes in terms of phytochemistry, toxicity, herbal processing, and pharmacology. It concludes by summarizing the changes in Fuzi’s compounds, toxicity, and pharmacological properties after processing.

By summarizing and analyzing the relevant literature, this review enriches the knowledge related to the processing of Fuzi and organizes the content related to the influence of processing methods on its pharmacological properties. Emphasizing the enhancement of Fuzi’s renal pharmacological effects through salt processing, the review thoroughly explores the mechanisms by which various processing methods affect its pharmacological effects.

## 2 Phytochemistry

**FIGURE 1:**
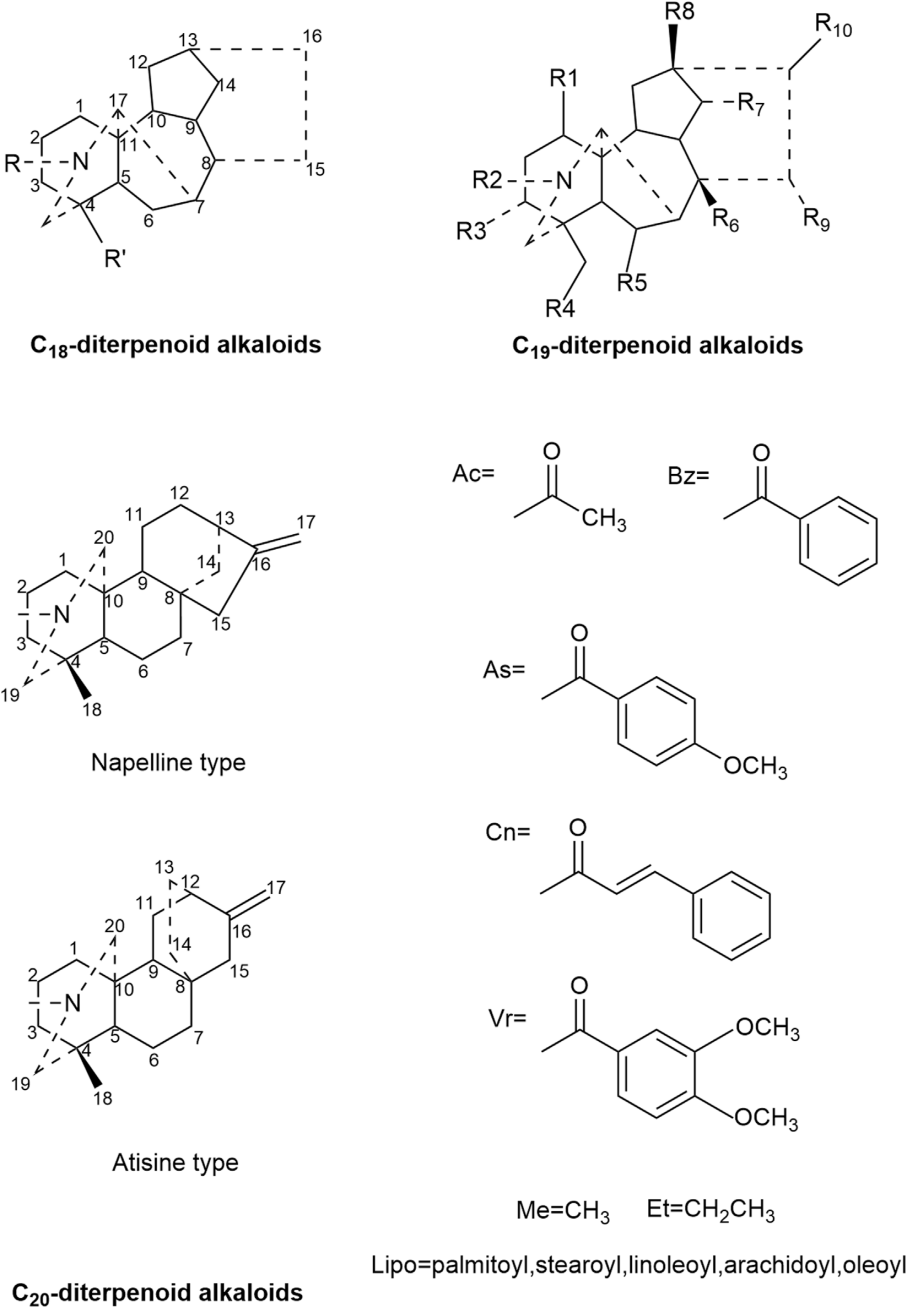
Three diterpenoid alkaloids’ skeleton and certain particluar groups.

In review, 122 compounds have been extracted and identified from *Aconitum carmichaelii* Debeaux (Zhang et al., 2022). Among these compounds, alkaloids are the principal active compounds responsible for the pharmacological activity, clinical efficacy, and even the toxicity of Fuzi. Consequently, these Fuzi alkaloids serve as indicator compounds in their quality evaluation. Based on the carbon atom count in their skeleton, these alkaloids are categorized as C_18_, C_19_, and C_20_ diterpenoid alkaloids (Wu et al., 2018; Gu et al., 2022) (Figure 1).

### 2.1 Fuzi’s alkaloids

Prior studies have demonstrated that Fuzi’s alkaloids possess a broad spectrum of pharmacological effects, including anti-inflammatory, anticancer, immunoregulatory, analgesic, and nephroprotective properties (Xu et al., 2021).

#### 2.1.1 C_19_ diterpenoid alkaloids

The primary bioactive and toxic compounds in Fuzi are the C19-diester-diterpenoid alkaloids (DDAs), predominantly comprising aconitine (AC), mesaconitine (MA), and hypaconitine (HA). C_19_ diterpenoid alkaloids are subdivided into various types, such as aconitine, lycoctonine, pyro-type, lactone-type, 7,17-seco-type, and rearranged-type, based on the specific oxygen-containing groups and structures at the C-7 position. *A. carmichaelii* primarily features alkaloids of the aconitine skeleton type, which exhibit diverse chemical properties due to different substituents at various positions within their fundamental skeleton (Wang et al., 2023). The structures of these C_19_ diterpenoid alkaloids in Fuzi are detailed in Table 1. Hydrolyzing the esters in Fuzi transforms DDAs into either monoester-diterpenoid alkaloids (MDAs) or ester-free amine-diterpenoid alkaloids (ADAs), significantly reducing their toxicity. For instance, the toxicity of MDA in rats decreases by 64–180 times compared to DDAs post-hydrolysis, without diminishing their pharmacological effects (Zhao et al., 2018). This reduction is why Chinese physicians have traditionally used decoctions and other processing methods to mitigate Fuzi’s toxicity, thus enabling its safer clinical application (Yang et al., 2023).

**TABLE 1:** Structures and names of the C_19_-diterpenoid alkaloids from Fuzi.

NO.	Chemical component	Molecular formula	R1	R2	R3	R4	R5	R6	R7	R8	R9	R10
1	Aconitine	C_34_H_47_NO_11_	OMe	Et	OH	OMe	OMe	OAc	OBz	OH	OH	OMe
2	Mesaconitine	C_33_H_45_NO_11_	OMe	CH3	OH	OMe	OMe	OAc	OBz	OH	OH	OMe
3	Hypaconitine	C_33_H_45_NO_10_	OMe	CH3	H	OMe	OMe	OAc	OBz	OH	OH	OMe
4	Talatizamine	C_24_H_39_NO_5_	OMe	Et	H	OMe	H	OH	OH	H	H	OMe
5	Isotalatizidine	C_23_H_37_NO_5_	OH	Et	H	OMe	H	OH	OH	H	H	OMe
6	Karacoline (Carmichaeline)	C_22_H_35_NO_4_	OH	Et	H	H	H	OH	OH	H	H	OMe
7	Neoline	C_24_H_39_NO_6_	OH	Et	H	OMe	OMe	OH	OH	H	H	OMe
8	Fuziline	C_24_H_39_NO_7_	OH	Et	H	OMe	OMe	OH	OH	H	OH	OMe
9	Isodelphinine	C_33_H_45_NO_9_	OMe	Me	H	OMe	OMe	OAc	OBz	H	OH	OMe
10	Benzoylmesaconine	C_31_H_43_NO_10_	OMe	Me	OH	OMe	OMe	OH	OBz	OH	OH	OMe
11	14-Acetyltalatisamine	C_26_H_41_NO_6_	OMe	Et	H	OMe	H	OH	OAc	H	H	OMe
12	Lipoaconitine	C_29_H_54_NO_7_	OMe	Et	OH	OMe	OMe	O-lipo	OBz	OH	OH	OMe
13	Lipomesaconitine	C_34_H_54_NO_7_	OMe	Me	OH	OMe	OMe	O-lipo	OBz	OH	OH	OMe
14	Lipohypaconitine	C_34_H_54_NO_7_	OMe	Me	H	OMe	OMe	O-lipo	OBz	OH	OH	OMe
15	Lipodeoxyaconitine	C_35_H_56_NO_7_	OMe	Et	H	OMe	OMe	O-lipo	OBz	OH	OH	OMe
16	Monoacetyltalizamine	C_26_H_41_NO_6_	OMe	Et	H	OMe	H	OH	OAc	H	H	OMe
17	Senbusine A	C_23_H_37_NO_6_	OH	Et	H	OMe	OH	OH	OH	H	H	OMe
18	Senbusine B	C_23_H_37_NO_6_	OH	Et	H	OMe	H	OH	OH	H	OH	OMe
19	Senbusine C	C_24_H_39_NO_7_	OH	Et	H	OMe	OMe	OH	OH	H	OH	OMe
20	Hokbusine A	C_36_H_59_NO_10_	OMe	Me	OH	OMe	OMe	OMe	OBz	OH	OH	OMe
21	Hokbusine B	C_34_H_55_NO_10_	OH	H	H	H	H	OH	OAc	H	H	OMe
22	Benzoylaconine	C_32_H_45_NO_10_	OMe	Et	OH	OMe	OMe	OH	OBz	OH	OH	OMe
23	Benzoylhypaconine	C_31_H_43_NO_9_	OMe	Me	H	OMe	OMe	OH	OBz	OH	OH	OMe
24	Neojiangyouaconitine	C_33_H_47_NO_9_	OMe	Et	H	OMe	OMe	Ome	OBz	OH	OH	OMe
25	Aldohypaconitine	C_33_H_43_NO_11_	OMe	CHO	H	OMe	OMe	OAc	OBz	OH	OH	OMe
26	Deoxyaconitine	C_34_H_47_NO_10_	OMe	Et	H	OMe	OMe	OAc	OBz	OH	OH	OMe
27	Beiwutine	C_33_H_45_NO_12_	OMe	Me	OH	OMe	OMe	OAc	OH	OBz	OH	OH
28	8-O-Cinnamoylneoline	C_33_H_45_NO_7_	OH	Et	H	OMe	OMe	OCn	OH	H	H	OMe
29	Karakanine	C_22_H_33_NO_4_	OMe	Et	H	H	H	OH	OH	H	H	OMe
30	14-O-Cinnamoylneoline	C_33_H_45_NO_7_	OH	Et	H	OMe	OMe	OH	OCn	H	H	OMe
31	14-O-Anisoylneoline	C_32_H_45_NO_8_	OH	Et	H	OMe	OMe	OH	OAs	H	H	OMe
32	14-O-Veratroylneoline	C_33_H_47_O_9_N	OH	Et	H	OMe	OMe	OH	OVr	H	H	OMe
33	Lipo-14-O-Anisoylbikhaconine	C_51_H_81_NO_10_	OMe	Et	H	Et	Et	O-lipo	OAs	OH	H	OMe
34	Lipoforesaconitine	C_28_H_51_AsNO_6_	OMe	Et	H	OMe	OMe	O-lipo	OAs	H	H	OMe
35	14-O-Acetyneoline	C_26_H_41_NO_7_	OH	Et	H	OMe	OMe	OH	OAc	H	H	OMe
36	Foresaconitine	C_35_H_49_NO_9_	OMe	Et	H	OMe	OMe	OAc	OAs	H	H	OMe
37	Crassicauline A	C_35_H_49_NO_10_	OMe	Et	H	OMe	OMe	OAc	OAs	OH	H	OMe
38	Lipoyunanaconitine	C_30_H_57_AsNO_6_	OMe	Et	OH	OMe	OMe	O-lipo	OAs	OH	H	OMe
39	8-Oet-14-Benzoylmesaconitine	C_36_H_59_NO_9_	OMe	Me	OH	OMe	OMe	OEt	OBz	OH	OH	OMe
40	Aconifine	C_34_H_47_NO_12_	OMe	H	OH	OMe	OMe	OH	OAc	OBz	OH	OMe
41	Aconine	C_25_H_41_NO_9_	OMe	Et	OH	OMe	OMe	OH	OH	OH	OH	OMe
42	Yunaconitine	C_35_H_49_NO_11_	OMe	Et	OH	OMe	OMe	OAc	OAs	OH	H	OMe
43	Chasmanine	C_25_H_41_NO_6_	OMe	Et	H	OMe	OMe	OH	OH	H	H	OMe
44	Foresticine	C_24_H_39_NO_7_	OMe	Et	H	OMe	OH	OH	OH	H	H	OMe
45	N-deethylaconine	C_25_H_44_NO_9_	OMe	H	OH	OMe	OMe	OH	OH	OH	OH	OMe
46	Beiwutinine	C_23_H_37_NO_9_	OMe	Me	OH	OMe	OMe	OH	OH	OH	OH	OH
47	Hypaconine	C_24_H_39_NO_8_	OMe	Me	H	OMe	OMe	OH	OH	OH	OH	OMe
48	Mesaconine	C_24_H_39_NO_9_	OMe	Me	OH	OMe	OMe	OH	OH	OH	OH	OMe
49	N-Ethylhokbusine B	C_24_H_37_NO_5_	OH	Et	H	H	H	OH	OAc	H	H	OMe
50	8-O-Ethylyunaconitine	C_35_H_51_NO_10_	OMe	Et	OH	OMe	OMe	OEt	OAs	OH	H	OMe
51	Oxonitine	C_33_H_43_NO_12_	OMe	CHO	H	OMe	OMe	OAc	OBz	OH	OH	OMe
52	(−)-(A-b)-8β-acetoxy-14α-benzoyloxy-N-Ethyl-13β,15α-dihydroxy-1α,6α,16β,18-tetramethoxy-19-oxo-aconitane	C_34_H_45_NO_11_	OMe	Et	CHO	OMe	OMe	OAc	H	OBz	OH	OH
53	(−)-(A-b)-8β-acetoxy-14α-benzoyloxy-N-ethyl-3α,10β,13β,15α-tetrahydroxy-1α,6α,16β,18-tetramethoxyaconitane	C_37_H_59_NO_11_	OMe	Et	OH	OMe	OMe	OAc	OH	OBz	OH	OH
54	(−)-(A-b)-14α-benzoyloxy-3α,10β,13β,15α-tetrahydroxy-1α,6α,8β,16β,18-pentamethoxy-N-methylaconitane	C_31_H_44_NO_11_	OMe	Me	OH	OMe	OMe	OH	OMe	OBz	OH	OMe
55	(−)-(A-c)-14α-benzoyloxy-3α,10β,13β,15α-tetrahydroxy-1α,6α,8β,16β,18-pentamethoxy-N-methylaconitane	C_25_H_42_NO_7_	OMe	H	H	H	H	OH	OAc	OH	OH	OMe
56	(−)-(A-b)-14α-benzoyloxy-N-ethyl-3α,10β,13β,15α-tetrahydroxy-1α,6α,8β,16β,18-pentamethoxyaconitane	C_34_H_48_NO_11_	OMe	Et	OH	OMe	OMe	OAc	OH	OBz	OH	OH
57	(−)-(A-b)-14α-Benzoyloxy-3α,10β,8β,13β,15α-Pentahydroxy-1α,6α,16β,18-Tetramethoxy-N-Methylaconitane	C_31_H_43_NO_11_	OMe	Me	OH	OMe	OMe	OH	OMe	OBz	OH	OMe
58	(−)-(A-b)-8β-acetoxy-14α-benzoyloxy-N-ethyl-3α,10β,13β-trihydroxy-1α,6α,16β,18-tetramethoxyaconitane	C_34_H_48_NO_11_	OMe	Et	OH	OMe	OMe	OH	OMe	OBz	OH	OMe
59	(−)-(A-b)-8β-acetoxy-14α-benzoyloxy-10β,13β,15α-trihydroxy-1α,6α,16β,18-tetramethoxy-N-methylaconitane	C_33_H_46_NO_11_	OMe	Me	H	OMe	OMe	OAc	OH	OBz	OH	OH
60	(−)-(A-b)-8β-Acetoxy-14α-benzoyloxy-N-ethyl-13β,15α-dihydroxy-1α,6α,16β,18-tetramethoxyaconitane	C_34_H_48_NO_10_	OMe	Et	H	OMe	OMe	OAc	H	OBz	OH	OH
61	(−)-(A-b)-14α-benzoyloxy-N-ethyl-8β,13β-dihydroxy-1α,6α,16β,18-tetramethoxyaconitane	C_32_H_46_NO_8_	OMe	Et	H	OMe	OMe	OH	OBz	OH	H	OMe
62	(−)-(A-b)-14α-benzoyloxy-N-ethyl-3α,8β,13β,15α-tetrahydroxy-1α,6α,16β,18-tetramethoxyaconitane	C_32_H_46_NO_10_	OMe	Et	OH	OMe	OMe	OH	OBz	OH	OH	OMe
63	(−)-(A-b)-8β,14α-dibenzoyloxy-N-ethyl-3α,13β,15α-trihydroxy-1α,6α,16β,18-tetramethoxyaconitane	C_38_H_48_NO_11_	OMe	Me	OH	OMe	OMe	OBz	H	OBz	OH	OH
64	(−)-(A-b)-14α-benzoyloxy-N-ethyl-13β,15α-dihydroxy-1α,6α,8β,16β,18-tetramethoxyaconitane	C_33_H_48_NO_9_	OMe	Et	H	OMe	OMe	OMe	OBz	OH	OH	OMe
65	(−)-(A-b)-14α-benzoyloxy-N-ethyl-8β,13β,15α-trihydroxy-1α,16β,18-trimethoxyaconitane	C_31_H_44_NO_8_	OMe	Et	H	OMe	H	OH	OBz	OH	OH	OMe
66	(−)-(A-b)-14α-benzoyloxy-N-ethyl-6α,8β,15α-trihydroxy-1α,16β,18-trimethoxyaconitane	C_31_H_44_NO_8_	OMe	Et	H	H	OMe	OH	OH	H	OBz	OH
67	(−)-(A-b)-14α-benzoyloxy-8β-ethoxy-N-ethyl-6α,15α-dihydroxy-1α,16β,18-trimethoxyaconitane	C_33_H_48_NO_8_	OMe	Et	H	H	OMe	OH	OEt	H	OBz	OH
68	(−)-(A-b)-14α-benzoyloxy-N-ethyl-8β,15α-dihydroxy-1α,16β,18-trimethoxyaconitane	C_31_H_44_NO_7_	OMe	Et	H	H	OMe	H	OH	H	OBz	OH
69	(−)-(A-b)-14α-benzoyloxy-N-ethyl-1α,8β,15α-trihydroxy-16β,18-dimethoxyaconitane	C_30_H_42_NO_7_	OH	Et	H	H	OMe	H	OH	H	OBz	OH
70	(−)-(A-b)-14α-benzoyloxy-N-ethyl-1α,8β,15α-trihydroxy-6α,16β,18-trimethoxyaconitane	C_31_H_44_NO_8_	OH	Et	H	H	OMe	OMe	OH	H	OBz	OH
71	(−)-(A-b)-14α-cinnamoyloxy-N-ethyl-1α,8β,15α-trihydroxy-6α,16β,18-trimethoxyaconitane	C_33_H_46_NO_8_	OH	Et	H	H	OMe	OMe	OH	H	OCn	OH
72	(−)-(A-b)-8β-acetoxy-14α-benzoyloxy-N-ethyl-15α-hydroxy-1α,6α,16β,18-tetramethoxyaconitane	C_34_H_48_NO_9_	OMe	Et	H	OMe	OMe	OAc	H	OBz	H	OH
73	Bullatine B	C_24_H_39_NO_6_	OH	Et	H	OMe	OMe	OH	OH	H	H	OMe

#### 2.1.2 C_20_ diterpenoid alkaloids

**FIGURE 2:**
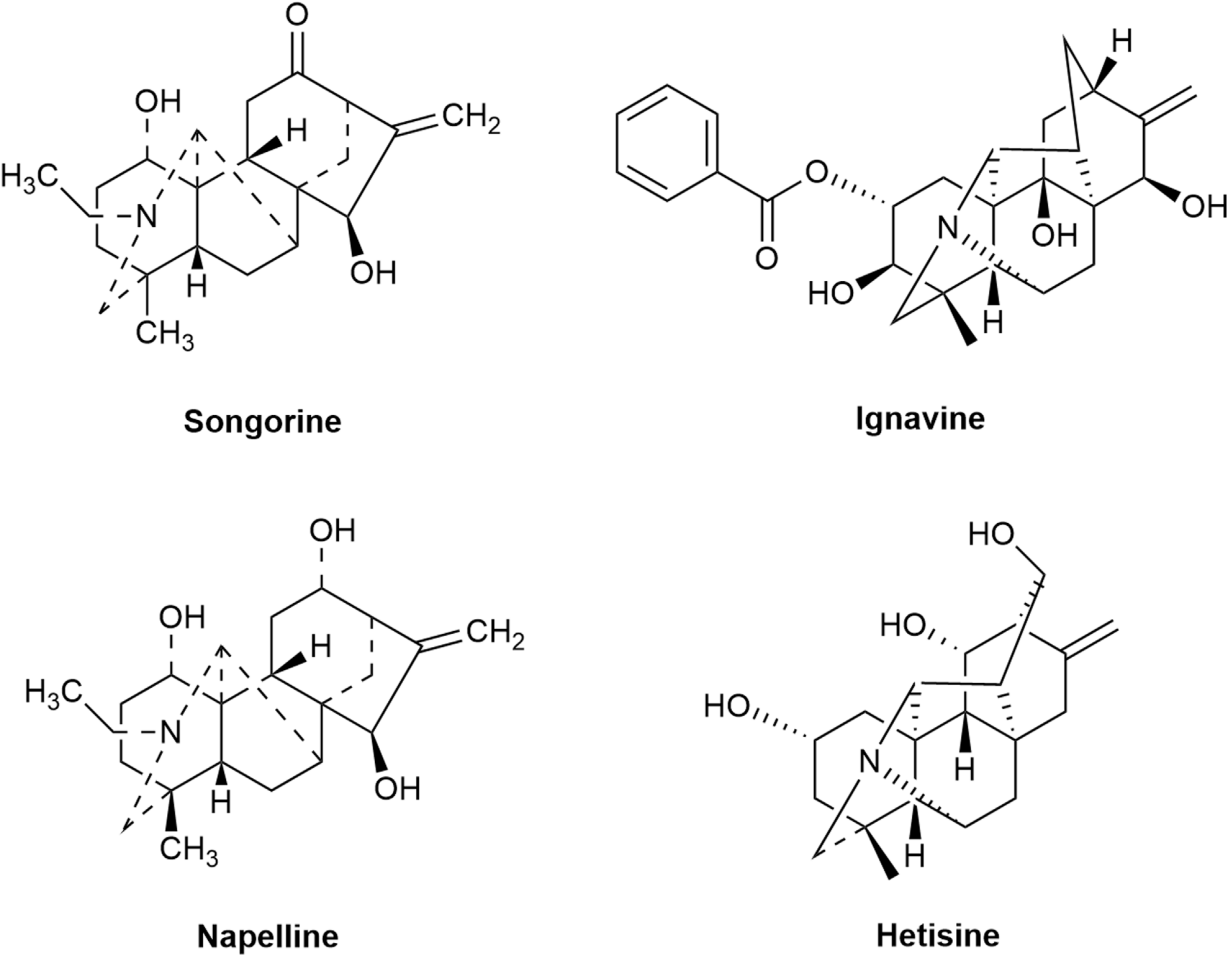
A part of C_20_-diterpenoid alkaloids from Fuzi.

C_20_ diterpenoid alkaloids represent the foundational diterpenoid alkaloids and are crucial as precursors for synthesizing various other diterpenoid alkaloids. These include atisine, denudatine, hetidine, hetisine, veatchine, napelline, and anopterine groups (Ren et al., 2017). Within Fuzi, the primary C_20_ diterpenoid alkaloids are songorine, ignavine, napelline, hetisine, *etc.,* (Figure 2).

#### 2.1.3 Other alkaloids

**FIGURE 3:**
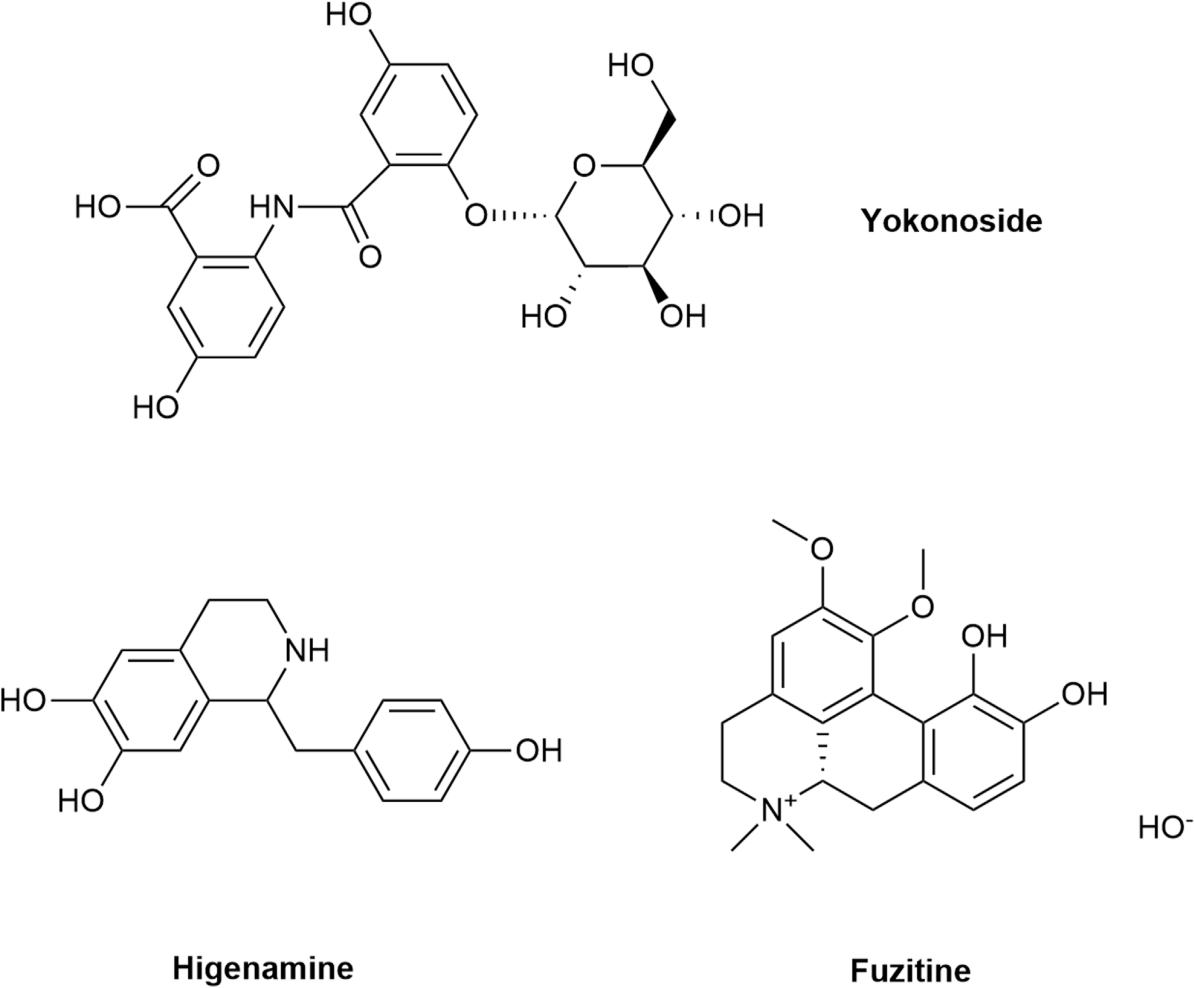
The main alkaloids contained in Fuzi, except for diterpenoid alkaloids.

Beyond diterpenoid alkaloids, other alkaloids such as yokonoside, higenamine, fuzitine, and aconicaramide have been identified. Their chemical structures are depicted in Figure 3.

### 2.2 Other compounds

In addition to alkaloids, Fuzi contains other compounds extracted from various plant parts, including flavonoids, polysaccharides, fatty acids, and ceramides. Fuzi’s polysaccharides, primarily comprising glucose, galacturonic acid, galactose, arabinose, and mannose, are noted for their anti-inflammatory effects, anticancer potential, immunomodulation, and cholesterol-reducing properties (Liu et al., 2019).

Fuzi flavonoids, mainly comprising flavanones and chalcones, are recognized for their antioxidant capabilities, inflammation reduction, cardiovascular health support, immune system modulation, and antibacterial, antiviral, and antifungal properties (Fu et al., 2022).

## 3 Toxicology

Despite its significant multi-systemic therapeutic effects, Fuzi’s narrow therapeutic window often yields severe toxicity in clinical practice, particularly affecting the heart, liver, and kidneys. To ensure drug safety, clinicians strictly regulate Fuzi’s dosage. This section examines the toxic compounds, symptoms, and mechanisms of Fuzi’s toxicity across different systems.

### 3.1 Toxic ingredients and clinical symptoms of poisoning

The primary toxic compounds in Fuzi are the DDAs, mainly including aconitine (AC), mesaconitine (MC), and hypaconitine (HC), with AC being exceptionally lethal at doses as low as 2 mg. HC, while being the most toxic, is present in the smallest quantity (Strzelecki et al., 2010). Symptoms of Fuzi’s poisoning include nausea, vomiting, palpitations, arrhythmias, muscle dysfunction, perioral paresthesia, respiratory distress, convulsions, gastrointestinal disturbances, and in severe cases, shock or coma. Death can occur due to ventricular arrhythmias. Currently, there is no direct antidote for Fuzi poisoning; treatment is primarily supportive and symptomatic (Chan et al., 2021).

### 3.2 Toxicity in various systems

#### 3.2.1 Cardiotoxicity

While being moderately beneficial for heart health, Fuzi could be cardiotoxic in high doses. AC affects cardiomyocytes by shortening the arrhythmic phase through enhancing intracellular Na^+^ flow and hastening cardiac membrane depolarization. At toxic levels, AC disrupts ion channels, promotes myocardial apoptosis, and may cause severe ventricular arrhythmias and myocardial damage (Zhou et al., 2021). Studies searching for potential tissue-specific biomarkers of Fuzi’s poisoning by myocardial lipidomics have identified alterations in lipid metabolites that participate in various metabolic pathways. Additionally, Cai et al. emphasized Fuzi’s dose-dependent cardiotoxic effects. These effects have been observed in histological cardiac damage and arrhythmias following overdose (Cai et al., 2013). Several studies have shown that AC induces Ca^2+^ overload and apoptosis in myocardial H9c2 cells by promoting the release and plasma membrane translocation of transient receptor potential cation channel subfamily V member 2 through the p38 MAPK signaling pathway (Yang et al., 2021). In an *in vitro* study on Fuzi’s cardiotoxic effects on zebrafish embryos and H9c2 cells, Li et al. exposed fertilized zebrafish embryos to AC. They confirmed that AC-induced cardiac dysfunction and apoptosis with an LD_50_ value of 7.92 μM and a 95% confidence interval ranging from 6.49 to 9.83 μM. Simultaneously, AC-induced intracellular Ca^2+^ oscillations increased the rate of apoptosis, inhibited the levels of TnT and Bcl-2 proteins, and promoted the upregulation of caspase 3 and Bax proteins in H9c2 cells after a 30-min treatment at a suitable concentration. These data confirmed that cardiac insufficiency and apoptosis induced by AC are related to the Ca^2+^ signaling pathway (Li et al., 2020). Furthermore, Fuzi water extracts and ethanol extracts exhibited cardiotoxicity by inducing apoptosis in cardiomyocytes by activating the PI3K/Akt/mTOR signaling pathway in rat myocardium tissue (Huang et al., 2018).

#### 3.2.2 Liver and kidney toxicity

Fuzi and its metabolites, when processed through the liver and kidneys, can induce liver and kidney damage. Studies using ultra-performance liquid chromatography-tandem mass spectrometry have noted a predominant distribution of toxic alkaloids in the liver and kidneys after long-term feeding of Fuzi preparations to mice, causing notable hepatic and renal damage. Conversely, these alkaloids were present at relatively low levels in the heart, brain, and blood (Ji et al., 2019). Hepatic and renal injury has been reported in some toxicity studies on rodents after single or long-term oral administration of Fuzi’s extracts. Symptoms of liver damage include elevated serum alanine aminotransferase and aspartate aminotransferase levels, edema, and necrosis, while kidney damage is characterized by increased serum creatinine (SCr) and blood urea nitrogen (BUN) levels, with histological evidence of lymphocyte infiltration and tissue atrophy (Zhou et al., 2016). Zhang et al. injected water extracts of Heishunpian (HSP) (a form of prepared Fuzi) intraperitoneally into mice; the results showed that these extracts could cause liver injury. Upon further investigation, the generation of hepatotoxic events can be attributed to the influence of toxic components on Th17 cell differentiation, Jak-STAT signaling pathway, and glutathione metabolism by modulating important targets such as AKT1, IL2, F2, GSR, and EGFR (Zhang et al., 2020). Sun et al. used metabolomics analysis to study the metabolic changes caused by toxic alkaloids of Fuzi in Wistar rats. The results showed that the maximum metabolic changes occurred six h after alkaloid treatment, leading to perturbations in renal tubular function. These changes lasted for about 24 h and tapered off after that time point (Sun et al., 2009). According to a TCM theory, Fuzi could be used to treat patients with the TCM “kidney-yang” deficiency pattern; modern research has also indicated that Fuzi has a nephroprotective effect that allows it to be used clinically in chronic kidney disease. A study on the different toxic responses of Baifupian (a form of prepared Fuzi) administration in healthy and hydrocortisone-exposed (mimicking the state of the kidney-yang deficiency pattern) rats showed that drug-induced toxic reactions were greatly attenuated in hydrocortisone-pretreated animals. Additionally, altered metabolic profiles involving oxidative phosphorylation, amino acid, and lipid metabolism featured alterations in phosphate, betaine, and phosphatidylcholine and may be associated with different toxic response profiles (Tan et al., 2013).

#### 3.2.3 Neurotoxicity

The central nervous system, susceptible to damage from toxins like AC, can exhibit acute neurotoxic effects, including motor blockade, cyanosis, asthenia, abdominal respiration, locomotor difficulty, and flaccid paralysis, often culminating in death (Li T. F. et al., 2016). The total fat-soluble alkaloids of Fuzi are neurotoxic to zebrafish; some researchers found that the toxicity may be related to the promotion of apoptosis and the effect on acetylcholinesterase activity through further experiments (Li et al., 2019). The potential mechanism of neurotoxicity resulting from Fuzi was initially elucidated by network pharmacology and molecular docking techniques. The results indicate that the neurotoxic mechanisms of Fuzi involve multiple targets and pathways. Notably, the MAPK signaling pathway and Akt protein-related pathway emerge as pivotal, significantly influencing cell membrane integrity, mitochondrial function, and neuronal apoptosis (An et al., 2022; Wang and Li, 2022).

#### 3.2.4 Embryotoxicity and reproductive toxicity

High doses of AC in zebrafish embryos induce malformations such as shortened body length, body curvature, pericardial edema, and impaired organ development, mediated by oxidative stress and mitochondrial apoptosis pathways (Xia et al., 2021). In male mice, Fuzi and its products have shown significant reproductive toxicity, evidenced by pathological changes in testicular tissue, reduced sperm count and viability, increased sperm abnormalities, and DNA damage, likely due to oxidative stress (Zhang et al., 2021).

## 4 Herbal processing

Given its unique pharmacological properties, Fuzi is extensively used in TCM. However, its narrow therapeutic window and significant toxicity often limit its clinical utility. Traditional processing techniques, honed over millennia, moderately mitigate these issues by altering the herb’s properties, flavor, and therapeutic channels, thus enhancing efficacy and reducing toxicity to ensure clinical safety. Additionally, the salt processing could particularly enhance the effect of Fuzi in kidney disease treatment.

### 4.1 Processing methods

#### 4.1.1 Thermal processing (called *huozhi* in Chinese)

*Huozhi*, or thermal processing, involves heat treatment of herbs without liquid adjuvants. This method, which includes stir-frying, roasting, and baking, varies by temperature, duration, and technique specifics. Historically, during the Han and Tang dynasties, this was the predominant method for preparing herbs (Liu et al., 2017).

Research by Deng et al. demonstrated that oven baking could decrease the concentration of toxic DDAs in Fuzi while preserving or increasing the levels of beneficial compounds, minimizing alkaloid loss compared to aqueous processing (Deng et al., 2012). Stir-frying with inert materials, like river sand, a common adjuvant, has been shown to preserve more active alkaloids and offer a simpler, more effective alternative to traditional methods (Peng et al., 2018).

#### 4.1.2 Aqueous processing (called *shuizhi* in Chinese)

*Shuizhi* involves processing herbs with water or liquid adjuvants (like salt water, vinegar, and wine), with or without heat. Zhang et al. noted that soaking Fuzi reduces its DDA content by 36.8% and MDAs by 25.5%, decreasing toxicity but potentially impacting therapeutic effectiveness (Zhang et al., 2017). Advanced techniques like pressurized steaming have been shown to maintain safety even at dosages of processed Fuzi much higher than typical clinical levels, underscoring the method’s reliability (Zhang, 2008). Furthermore, innovations like treatment with a saturated solution of Ca(OH)_2_, extended cooking times, and high-pressure cooking have been effective in reducing toxic diterpenoid alkaloid content significantly while enhancing the concentrations of less toxic forms, thus optimizing the safety and effectiveness of processed Fuzi (Yu et al., 2023).

#### 4.1.3 Innovative methods

In addition to the two previously mentioned commonly used processing methods of Fuzi, the integration of modern technology with traditional processes has introduced novel methods such as microwave processing, high-pressure treatment, and microbial fermentation (Yang et al., 2019). Microwave processing, characterized by high temperature, rapid heating, and a sterilizing effect, efficiently hydrolyzes toxic alkaloids in raw Fuzi, enhancing the solubility and stability of its active compounds, which improves the safety, efficacy, and medicinal value of the herb (Li et al., 2022).

### 4.2 Mechanisms of enhanced efficacy and reduced toxicity

#### 4.2.1 Hydrolysis reaction

**FIGURE 4:**
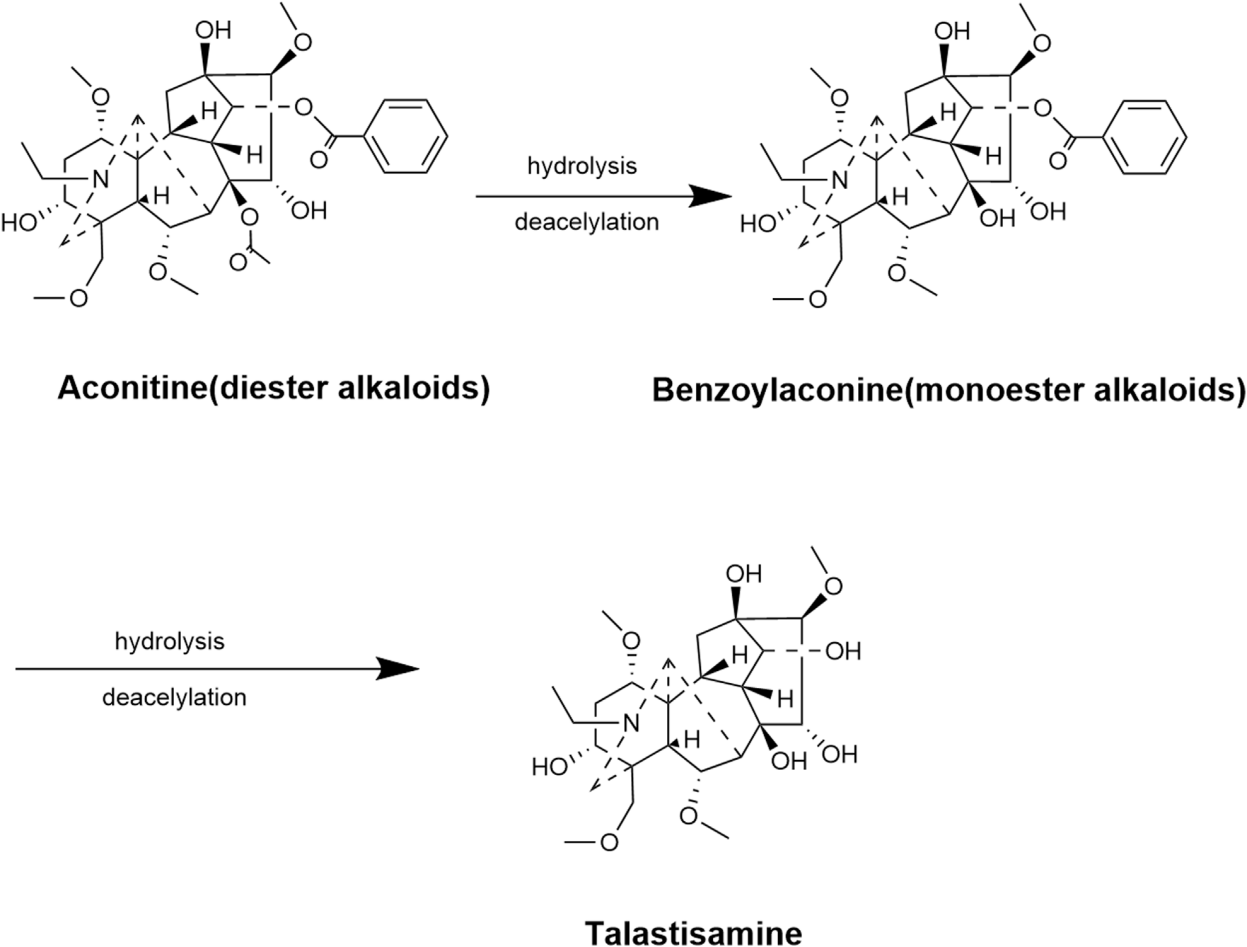
Hydrolysis reaction of aconitine.

At the appropriate temperature, the alkaloids in Fuzi react with water, undergoing hydrolysis to produce new components that modify the efficacy and toxicity of the drug. Modern pharmacological studies have identified a significant number of DDAs in Fuzi, which are pivotal to its therapeutic effects and toxicity. Structurally, Fuzi contains DDAs, MDAs, and non-esterolamine alkaloids. DDAs, such as AC, exhibit substantial pharmacological activity but high toxicity, constituting the primary toxic compounds. MDAs display reduced toxicity (1/50 to 1/500 of AC) and significant pharmacological effects, making them crucial in clinical applications. Non-esterolamine alkaloids possess even lower toxicity (1/2,000 to 1/4,000 of AC) but weaker pharmacological activity (Wei et al., 2019). AC, a highly toxic DDA, becomes unstable at high temperatures, with two ester bonds undergoing hydrolysis to form benzoyl monomer alkaloid (benzoylaconine), which has approximately 1/200 the toxicity of AC. Further heating leads to the hydrolysis of benzoylaconine into the alcoholamine AC alkaloid with the loss of the benzoyl group (Sun et al., 2016) (Figure 4). Herbal processing techniques, including decoction, steaming, and microwave processing, are employed to mitigate Fuzi’s toxicity through hydrolysis, thereby enhancing its clinical safety and efficacy. A study by Zhang et al. showed that the concentration of DDAs in crude Fuzi’s aqueous extract decreased rapidly during the initial decoction phase and stabilized at a lower level after 4 h, while MDA levels initially increased, peaking between 4 and 6 h. This pattern aligns with the traditional processing principle of boiling Fuzi for 4–6 h (Zhang et al., 2015).

#### 4.2.2 Replacement reaction

A replacement reaction typically involves a simple substance reacting with a specific compound to yield new elements and compounds. Similar substitution reactions occur during the herbal processing of Fuzi, where certain groups in the herb’s main compounds are substituted by other small organic molecules, ultimately altering the drug’s therapeutic efficacy (Li et al., 2021). Concocting Fuzi with Radix Glycyrrhiza (RG) involves the replacement reaction; *Jing Yue’s complete work* published in the Ming Dynasty summarized the detoxifying principles of concocting Fuzi with RG using Chinese medicine theories. It introduced the specific method of utilizing RG to concoct Fuzi, “If you want to use prepared Fuzi urgently, wrap crude Fuzi with thick paper, pour RG soup over it, then simmer or bake, until it is soft, cut, then wrap with paper and pour RG soup, bake it again, repeat the above steps when it’s cooked thoroughly” (Chen et al., 2014). Fatty acids in RG undergo a lipid exchange reaction with the DDAs in Fuzi and their degradation products. This reaction ultimately produces less toxic lipid alkaloids; simultaneously, a small amount of DDAs forms an insoluble precipitate together with glycyrrhizinic acids in RG, which reduces the DDA content, thus diminishing toxicity (Cai et al., 2012). Processing Fuzi with RG significantly alters its chemical profile, decreasing the concentration of DDAs by about 98%, while increasing MDAs by approximately 0.6 times. HPLC-MS analyses have tentatively identified new components in the processed product, such as liquiritin and ononin (Guo et al., 2015).

#### 4.2.3 Destruction and loss of alkaloids

The processing of Fuzi often involves washing, soaking, moistening, and rinsing. Despite not involving chemical reactions, the processing effectively reduces toxicity by causing the destruction and loss of alkaloids. These steps are crucial in Fuzi’s concoction as long as they do not cause excessive alkaloid loss.

### 4.3 Detoxifying effects of different processing methods

Advancements in processing technology have diversified the methods applied to Fuzi, extending beyond traditional techniques. While different methods can reduce Fuzi’s toxicity to varying extents, a key consideration is their ability to diminish toxic substances without compromising the retention of active pharmacological compounds.

According to the 2015 edition of the Chinese Pharmacopoeia, the allowable content of DDAs (the sum of AC, MC, and HC) in Heishunpian and Baifupian—two common forms of processed Fuzi—should not exceed 0.020%. Similarly, the content of monoester alkaloids (the sum of benzoylaconine, benzoylmesaconine, and benzoylhypaconine) should not be less than 0.010%. Additionally, regulations in Sichuan Province mandate that the content of DDAs in raw sliced Fuzi should be 0.10%–0.20% (Zan et al., 2019; Gu et al., 2022). Table 2 illustrates the variations in toxic and active components of Fuzi following different processing methods (Su et al., 2010; Yang et al., 2014; Xu et al., 2017; Ye et al., 2019; Xia et al., 2021; He et al., 2023).

**TABLE 2:** Detoxifying effects of different processing methods.

Processing method	Ingredient	Processing time	Unit (content)	Aconitine		Mesaconitine		Hypaconitine		Diester alkaloids	
				Unprocessed	Processed	Unprocessed	Processed	Unprocessed	Processed	Unprocessed	Processed
Steaming	raw Fuzi	90 min	mg/kg	184.03	0.45	736.29	0.47	829.53	3.84	1749.85	4.77
Baking	raw Fuzi	60 min	mg/kg	184.03	9.07	736.29	27.46	829.53	48.25	1749.85	84.78
Decocting	raw Fuzi	8 h	mg/kg	184.03	0.39	736.29	0.52	829.53	2.00	1749.85	2.91
Stir-frying (with sand)	raw Fuzi	—	mg/mL	0.0195	0.0048	0.0649	0.0059	0.1872	0.0268	0.0863% (proportion of sample content)	0.0119% (proportion of sample content)
Fermenting Processing with adjuvants	raw Fuzi	—	mass fraction (%)	0.0670%	0.0522%	0.2750%	0.0633%	0.0550%	0.0291%	0.3970%	0.1446%
Processing with Radix Glycyrrhizae and Black Soya Bean	salt-processing Fuzi	—	mass fraction (%)	0.0038%	—	0.018%	—	0.017%	0.0013%	0.039%	0.0013%
Processing with ginger juice	raw Fuzi	—	mg/g	142.4926	50.1496	298.5440	27.0973	473.6812	109.2593	914.7178	186.5062
Processing by microwave	raw Fuzi	—	mass fraction (%)	—	—	—	—	—	—	—	0.004%–0.016%

Benzoylaconine		Benzoylmesaconine		Benzoylhypaconine		Monoester alkaloids		References
Unprocessed	Processed	Unprocessed	Processed	Unprocessed	Processed	Unprocessed	Processed	
15.67	145.25	137.02	878.91	22.79	632.14	175.48	1,656.30	Yang et al. (2014a)
15.67	92.61	137.02	594.39	22.79	335.99	175.48	1,022.99	Yang et al. (2014a)
15.67	145.86	137.02	807.34	22.79	584.59	175.48	1,537.79	Zhang et al. (2015a)
0.0344	0.0735	0.0934	0.1641	0.0310	0.0970	0.0415% (proportion of sample content)	0.0785% (proportion of sample content)	Ye et al. (2019)
—	—	—	—	—	—	—	—	Su et al. (2010)
0.00032%	0.0013%	0.0046%	0.00820%	0.00070%	0.0035%	0.0053%	0.013%	Guo et al. (2015)
194.2307	16.2768	17.4816	42.0960	68.8040	22.1509	280.5163	80.5237	Xu et al. (2017)
—	—	—	—	—	—	—	0.071–0.166%	He et al. (2023)

### 4.4 Various changes in Fuzi after processing

#### 4.4.1 The changes in compounds

The processing initially induces alterations in the compounds of Fuzi; these compounds serve as the foundation for its toxicity and pharmacological effects. Using HPLC measurements to analyze the changes in the chemical composition of Fuzi after sand scalding, the results indicated a decrease in the content of DDAs from 0.0675% to 0.0015%, representing a reduction of approximately 98%. Conversely, the content of MDAs increased approximately threefold from 0.0141% to 0.0382% (Chen et al., 2016). In a separate study, the UPLC-Q-TOF-MS/MS method was employed to identify compounds that exhibited variations before and after low-temperature baking of raw Fuzi. By utilizing variable importance in the projection (VIP), 44 potentially differentiated compounds were identified (Ye et al., 2021). Through employing the UHPLC-Q-TOF/MS technique to gather data on the chemical compound groups during the decoction of Fuzi, Li et al. discovered that the compositional changes during decocting primarily involved the conversion of DDAs to MDAs, and subsequently to aconine alkaloids. Notably, the transformation of MDAs to aconine alkaloids was predominant. Furthermore, ester alkaloids such as karakoline, songorine, and fuziline exhibited negligible changes during prolonged heating, attributed to their excellent thermal stability (Li R et al., 2016).

In addition to studies on compositional changes following a single processing method, research involving multiple processing techniques has been conducted. Heishunpian and Baifupian, two common forms of processed Fuzi involving thermal and aqueous treatments, were utilized for compositional comparisons with unprocessed Fuzi using chemical UPLC-MS profiling and multivariate classification techniques. A total of 18 compounds, including one veatchine alkaloid, seven ADAs, six DDAs, three MDAs, and one lipo-diterpenoid alkaloid (LDA), were identified in this study. While the two processed products exhibited a significant decrease in the content of highly compounded DDAs compared to raw Fuzi, there was an increase in the content of catabolized low-toxicity derivatives such as MDAs, ADAs, and LDA (Sun et al., 2019).

#### 4.4.2 The changes in toxicity

Processing-induced changes in toxic alkaloids within raw Fuzi undoubtedly impact the toxicity of the processed Fuzi. Ren et al. investigated the alterations in the content of Aconitum alkaloids in Fuzi following various processing methods using DESI-MSI and UHPLC-QTOF MS. The results revealed that the content of less toxic MDAs and NAs, converted from the more toxic DDAs, increased in all processed products. Additionally, the content of benzoylaconine increased post-processing, indicating enhanced pharmacological activity and reduced toxicity (Ren et al., 2023). Furthermore, processed Fuzi can elevate testosterone, superoxide dismutase (SOD), glutathione (GSH), and catalase (CAT) levels while decreasing malondialdehyde levels in serum compared to raw Fuzi, thereby mitigating the reproductive toxicity and genotoxicity of Fuzi. The protective mechanism of processing to alleviate Fuzi toxicity may be associated with increased testosterone levels and reduced oxidative stress (Zhang et al., 2021). The processing of Fuzi with RG represents a classical method capable of reducing the toxicity of raw Fuzi. The detoxification principle of this method involves decreasing the content of DDAs through replacement reactions. However, a study found that co-administration of Fuzi and RG could attenuate significant metabolic changes in several amino acids, organic acids, and ketone bodies induced by Fuzi alone in rats. This suggests that reducing toxicity at the metabolic level through various pathways, including energy metabolism and the synthesis and degradation of ketone bodies, may represent another potential detoxification mechanism of Fuzi processed with RG (Sun et al., 2016).

#### 4.4.3 The changes in pharmacological effects

Processing alters the composition of Fuzi, leading to variations in pharmacological effects. Heishunpian is a processed Fuzi product involving multiple complex processing steps. To investigate the impact of each step on its anti-inflammatory and analgesic effects, a study employed a “step knockout” strategy to assess the efficacy of different samples. The findings revealed that ibuprofen and Heishunpian exhibited the highest efficacy in the hot plate test, while ibuprofen and the sample without rinsing were the most effective in the acetic acid torsion test. The differences in efficacy may be attributed to the varying effects of different steps on the content of active analgesic components, such as mesaconine, aconine, 3-deoxyaconine, delbruine, and asperglaucide (Xue et al., 2024). Processing influences the pharmacological effects of Fuzi and its therapeutic mechanism in specific diseases. Tong et al. compared the differences in expressed proteins between unprocessed Fuzi and its processed product (Yinfupian, characteristic decoction pieces for the Yang deficiency syndrome) inducing Yang deficiency syndrome in rats. They found that raw Fuzi primarily targeted muscle contraction-related proteins, closely linked to inflammation and myocardial injury. Conversely, the processed products primarily affected mitochondrial proteins, closely associated with adenosine triphosphate energy metabolism (Tong et al., 2021).

### 4.5 The theory of “salt processing leading drug into kidney”

The 2020 edition of the Chinese Pharmacopoeia highlights that treating crude Fuzi with a salt solution is essential. This standard draws on the extensive experience of TCM practitioners developed through long-term clinical practice. The exact mechanism behind Fuzi’s salt processing remains elusive; however, the theory of “salt processing directing drugs to the kidney” provides a partial explanation.

#### 4.5.1 Salt processing

Salt processing, a classical pharmaceutical technique in TCM, involves using salt as an auxiliary ingredient. Salt, characterized by its flavor and cold nature, is known for clearing heat, cooling blood, softening and dispersing masses, and moistening dryness. After salting the Chinese medicine, it will mainly enter the kidney meridian. Historically, *Leigong’s Treatise on Preparations* from the Northern and Southern Dynasties first documented the use of salt to process various medicines (Liu et al., 2023). Modern medical theory acknowledges that salt, primarily composed of NaCl along with traces of MgCl_2_, CaCl_2_, KCl, and NaI, is crucial for maintaining normal osmotic pressure in human tissues. Salt enhances gastric juice secretion and protein absorption and has a diuretic effect (Zhou et al., 2021). Initially, during the Sui Dynasty, salt processing was used to enhance the curative effect and reduce the toxicity of Fuzi, with its antiseptic and preservative qualities being significant under less advanced conditions. Presently, salt processing comprises salt washing, salt infiltration, salt infiltration heating, and salt stir-frying, with saltwater frying and steaming being the most common (Hou et al., 2023).

#### 4.5.2 Influence of salt processing on the renal action of drugs

Rooted in the foundational TCM five-phase theory, which posits that “five flavors enter the five viscera,” saltiness is associated with the kidney. Despite the differing definitions of internal organs in TCM and modern medicine, certain references can still be drawn. The physiological characteristics of the kidney, particularly its role in Na^+^ reabsorption, enable many active ingredients in salt-processed drugs to be absorbed by the kidneys along with Na^+^ reabsorption in clinical application (Wei et al., 2019).

Although many scholars have mentioned in their studies that salt processing enhances the effects of drugs on the kidney, there is a lack of research on the effects of salt-processed Fuzi on the kidney. However, insights might be drawn from studies on other salt-processed drugs or compound medicines (Bai et al., 2023). For instance, research using NMR techniques has shown that salt processing significantly alters the chemical composition of Alismatis rhizoma (ALR), commonly used for treating kidney diseases, by promoting dehydration, deacetylation, and oxidation (Han et al., 2016). HPLC studies conducted by Cao et al. have confirmed increases in specific bioactive components in salt-processed ALR, enhancing its diuretic effect (Cao et al., 2016).Suoquan pill composed of salt-processed Yizhiren (*Alpiniae oxyphyllae*) could significantly reduce the serum levels of BUN and creatinine in the kidney-yang deficiency model mice compared with Suoquan pill composed of crude Yizhiren and have a more pronounced effect on the repair of renal injury in model mice. The result validates the science and correctness of using salt-processed Yizhiren instead of raw Yizhiren in the Suoquan pill (Shuai et al., 2018).

## 5 Pharmacology

### 5.1 Pharmacodynamics of Fuzi on the kidney

Fuzi, a TCM, is known for its pungent and sweet flavors, extreme heat nature, and toxicity. It is associated with the heart, kidney, and spleen meridians. Clinically, Fuzi is a primary ingredient in treatments for various kidney ailments, such as acute and chronic glomerulonephritis and nephrotic syndrome (Jiang et al., 2023). Modern pharmacological research suggests that its nephroprotective, anti-inflammatory, and immunomodulatory properties contribute to these effects (Table 3).

**TABLE 3:** Pharmacological effects of Fuzi on the kidney.

Pharmacology	Extract/Chemical components/Decoction	Experimental animals/cells	Model establishment	Experimental dose	Time of administration	Effects	References
Nephroprotective effect							
Antioxidant effect	Fuzi water extract	mouse	Intragastric administration of aristolochic acid	5 g/kg/d	2 weeks	↓renal index, ↓SCr, ↓BUN, ↓UA ↑γ-GT	Du et al. (2012)
	modified Fuzi Lizhong Decoction	rat	Gavage administration of aristolochic acid	10 mL/kg	3 weeks	↑SOD ↓MDA	Shi et al. (2011)
	Fuzi polysaccharides	NRK-52E cells, rat	Treated with H2O2, Intraperitoneal injection of cisplatin	5000, 2,500, 1,250 μg/mL 200, 400, 800 mg/kg	24 h, 10 days	↓cell apoptotic rate ↑GSH, ↑GPX-4, ↓MDA, ↓4-HNE	Tian et al. (2022)
Interventional effect on apoptosis and autophagy	Fuzi water extract Fuzi polysaccharides	HMCs	Treated with 10 μg lipopolysaccharides	100 μL	24 h	↑ Bax, ↑ caspase-8, ↑caspase-3 ↓Cyclin E, ↓CDK2	Li et al. (2022b)
	modified Fuzi Lizhong Decoction	rat	Blocked left and right renal arteries with arterial clips	10 mL/kg	7 days	↓SCr, ↓BUN, ↓MDA, ↓caspase-3, ↓PARP, ↓Bax ↑SOD	Lan et al. (2020)
	Mahuang Fuzi and Shenzhuo Decoction	rat	Injected with anti-Fx1A Serum	1 mL/100 g	12 weeks	↓24 hUTP, ↓CHO, ↓TG, ↓nephrin, ↓IgG, ↓LC3-II, ↓p62, ↓β-catenin, ↓GSK-3β	Gao et al. (2022)
	Dahuang Fuzi Decoction	rat	Administered 2% adenine	2.5 g/kg	3 weeks	↓proteinuria, ↓NAG, ↓BUN, ↓SCr, ↓Bax, ↓caspase-3, ↓TGF-β1, ↓p-JNK ↑ Bcl-2	Tu et al. (2014)
Promotion of metabolic waste excretion	Fuzi water extract	mouse	Gavage administration of aristolochic acid Gavage administration of adenine	3,10 g/kg	5 weeks 4 weeks	↓BUN, ↓LD, ↓LDH, ↓kidney coefficient ↑sperm counts, ↑renal protein contents	Fan et al. (2011)
	Fuzi water extract	rat	Intravenous injection of adriamycin	3, 6, 9 g/kg	8 weeks	↓urinary protein, ↓TC, ↓TG, ↓BUN, ↓SCr ↑TP, ↑ALB	Yang and Wang (2010)
Anti-inflammatory and antifibrotic effects	Fuzi water extract	mouse	administered adenine by oral gavage	1 g/kg	5 days	↓Scr, ↓BUN, ↓NAG, ↓IL-1β, ↓IL-6 ↓TNF-α, ↓Ccl2, ↓Tgfb1, ↓Col1a1	Yang et al. (2021b)
	Fuzi and Ganjiang extraction	BV2 cells	Treated with LPS	400 μg/mL	—	↓IL-6, ↓TNF-α, ↓ROS, ↓NO ↓PGE2, ↓iNOS, ↓COX2	Yang et al. (2021a)
	ShenFu Decoction	rat	Administered 2.5% adenine	7.56 g, 3.78, 1.89 g/kg	4 weeks	↓TGF-β1, ↓CTGF, ↓TNF-α ↑Smad7	Yuan (2020)
Immunomodulatory effect	Aconitine	mouse	Intraperitoneal injection of 0.5 mL pristane	25, 75 μg/kg	9 weeks	↓blood leukocyte counts ↓serum anti-dsDNA antibody ↓IgG deposit, ↓PGE2, ↓IL-17a, ↓ IL-6	Li et al. (2017)
	Fuzi water extract	rat	Injected with hydrocortisone sodium succinate	12 g/kg	7 days	↓IFN-γ ↑Na^+^-K^+^-ATPase ↑Mg^2+^-ATPase, ↑SDH	Li et al. (2021b)
	Fuzi polysaccharides	mouse	Intraperitoneal injections of cyclophosphamide	200, 100, 50 mg/kg	7 days	↑NO, ↑IFN-γ, ↑spleen index, ↑thymus index, ↑proliferation of spleen cells and peritoneal macrophages	Fu et al. (2018)
	Fuzi neutral polysaccharide	mouse	Inoculated subcutaneously with H22 liver cancer cells	100, 200 mg/kg	7 days	↓IL-10, ↓TNF-α ↑IL-1β, ↑IL-6	Hu et al. (2023)

#### 5.1.1 Nephroprotective effects

Various animal models of nephropathy have elucidated potential mechanisms behind Fuzi’s renal protective effects. These include antioxidative stress, regulation of apoptosis and autophagy, and enhancement of metabolic waste excretion.

##### 5.1.1.1 Antioxidant effects

Aristolochic acid nephropathy (AAN), characterized by acute and chronic tubulointerstitial lesions, results in acute and chronic renal failure and tubular acidosis. Previous studies have suggested that the nephrotoxicity of aristolochic acid may be related to lipid peroxidation and DNA oxidative damage which result from elevated levels of reactive oxygen species *in vivo* (Du et al., 2012). A Study on the AAN rat model has shown that the modified Fuzi Lizhong Decoction (mFLD) increases SOD activity in renal tissues, reduces malondialdehyde (MDA) levels, and alleviates renal pathology, underscoring Fuzi’s antioxidant properties (Shi et al., 2011). Refined fucose polysaccharide (RFP), extracted from Aconitum carmichaelii’s lateral root, exhibits antioxidant activity by enhancing the clearance rates of various free radicals and reducing lipid peroxidation. Taking it a step further, Tian et al. uncovered that RFP could reduce cell membrane lipid peroxidation in mice with cisplatin-induced acute kidney injury by increasing the levels of GSH and glutathione peroxidase-4 and lowering the levels of malondialdehyde (MDA) and 4-hydroxynonenal (Chen et al., 2022; Tian et al., 2022).

##### 5.1.1.2 Interventional effects on apoptosis and autophagy

Apoptosis and autophagy are both forms of programmed cell death; many studies indicate that Fuzi and its components have an intervening effect on them. For example, aqueous extracts of Aconiti Lateralis Radix and its polysaccharides inhibit mesangial cell proliferation via the PI3K/AKT/mTOR pathway and induce apoptosis by modulating key proteins (such as Cyclin E/CDK2, Bax, caspase-8, and caspase-3) involved in the cell cycle and death pathways in a study discussing the underlying therapeutic mechanisms of mesangial proliferative glomerulonephritis (MesPGN) (Li et al., 2022). Ischemia-reperfusion is one of the most common causes of acute kidney injury; current research suggests that the underlying mechanism is the massive production of oxygen free radicals after reperfusion, which leads to extensive oxidative stress injury and then induces apoptosis. Lan et al. explored the protective effect of the prophylactic administration of mFLD on the ischemia-reperfusion injury rat model; the results suggested that it may be related to the antioxidant stress response, modulation of apoptotic pathways through inhibiting the expression of Caspase-3, PARP, and Bax proteins (Lan et al., 2020). During the investigation into potential mechanisms of Mahuang Fuzi and Shenzhuo Decoction (MFSD) in treating membranous nephropathy (MN), researchers observed significant reductions in proteinuria and podocyte injury in passive Heymann nephritis rats (a classical MN model) following MFSD treatment. This corresponded with improvements in renal pathology. Analysis of the study suggests that modulation of autophagy in podocytes and inhibition of the Wnt/β-catenin pathway could be potential targets (Gao et al., 2022). Furthermore, given that Dahuang Fuzi Decoction (DFD) is a traditional formula clinically proven for treating chronic kidney disease (CKD) in China, Tu et al. investigated its therapeutic effects on the adenine-induced renal injury rat model to elucidate the *in vivo* therapeutic mechanism. The findings revealed that DFD mitigated apoptosis by downregulating the expression of Bax and cleaved caspase-3 proteins while enhancing Bcl-2 protein expression. Furthermore, it attenuated renal tubular epithelial apoptosis by suppressing the activation of the TGF-β1-JNK pathway (Tu et al., 2014; Ta et al., 2023).

##### 5.1.1.3 Promotion of metabolic waste excretion

Fan et al. investigated the impact of Fuzi on lactic acid (LD) metabolism in two different mouse models of CKD. Both groups of mice exhibited varying degrees of LD acidosis. Following the administration of Fuzi’s aqueous decoction, serum or organ LD levels decreased, while the activity of lactate dehydrogenase (LDH) significantly increased in both groups. These findings suggest that Fuzi exerts a nephroprotective effect by modulating LD metabolism. The underlying mechanism may involve Fuzi’s ability to reduce LD production or enhance LD excretion, consequently diminishing LD accumulation, correcting acidosis, and improving energy metabolism (Fan et al., 2011). Moreover, in a study on the therapeutic effect of Fuzi on adriamycin-induced nephropathy in rats, significant reductions in SCr and BUN were observed in the treatment group compared to the model group, reflecting Fuzi’s ability to promote the excretion of serum urea nitrogen and creatinine (Yang and Wang, 2010).

#### 5.1.2 Anti-inflammatory and antifibrotic effects

Renal damage typically involves inflammatory and fibrotic responses. When various factors damage renal intrinsic cells, endothelial cytokines (such as TNF-α and IL-1) become activated. This activation prompts the recruitment of diverse inflammatory cells from the bloodstream, which infiltrate the membranes, capillaries, and renal mesangial area, subsequently releasing a plethora of inflammatory mediators. Following a cascade of inflammatory reactions and cytokine activity, renal intrinsic cells release fibrogenic factors, transforming into myofibroblasts. These myofibroblasts synthesize and secrete substantial amounts of collagen fibers that resist degradation, yielding an imbalance in extracellular matrix formation and degradation, ultimately forming a scar. These alterations culminate in renal histoarchitectural damage, renal interstitial fibrosis, and a progressive decline in renal function.

Yang et al. found that the main active ingredients of Fuzi, such as AC, HC, and neoconitine, could reduce SCr, BUN, and urinary N-acetyl-β-D-glucosidase levels and decrease inflammatory and fibrotic factors (IL-1β, IL-6, TNF-α, Ccl2, Tgfb 1, and Col1a1) in mice with adenine-induced CKD (Yang et al., 2021). Another study also showed that the Chinese herb couple, Fuzi and Ganjiang, effectively reduced the production of pro-inflammatory mediators (IL-6, TNF-α, ROS, NO, and PGE2) and inhibited the expression of iNOS and COX2, which was associated with the inhibition of NF-κB/activation of the Nrf2/HO-1 signaling pathways (Yang et al., 2021). After comparing renal histopathological changes in an adenine-induced CKD rat model treated with Shenfu Decoction (SD), Yuan et al. hypothesized that SD might inhibit the irregular release of fibrogenic factors and reduce inflammatory cell infiltration and fibrous tissue proliferation through the TGF-β1/Smads signaling pathway, thus exerting an anti-fibrotic effect on renal interstitium (Yuan, 2020).

#### 5.1.3 Immunomodulatory effect

Immunoregulatory mechanisms are a driving force in the pathogenesis of many kidney diseases. Moreover, immunosuppressants are commonly used in the treatment of kidney diseases (Wang and Zhang, 2017). One of the common syndrome types of low immune function in TCM is kidney-yang deficiency. Therefore, warming and tonifying kidney yang is an important rule to improve human immune function; Fuzi is one of the prime choices for reinforcing kidney yang (Yuan, 2018).

Many secondary kidney diseases are caused by autoimmune disorders. For instance, lupus nephritis is one of the most serious complications of systemic lupus erythematosus (SLE). Li et al. observed the pathological lesions of lupus nephritis induced by phytane in mice; the results showed that AC reduced the blood leukocyte count, lowered the serum anti-dsDNA antibody level, and improved the health status, thus reducing the damage to the organism. In conclusion, AC has a strong immunosuppressive effect and may inhibit the activity and progression of SLE (Li et al., 2017). Hydrocortisone sodium succinate injection-induced cold immunocompromise and energy metabolism disorders in rats are significantly ameliorated via Fuzi aqueous extract (Li et al., 2021).

Fuzi’s impact on immune functions is particularly significant in conditions where immunity influences kidney disease progression. Recent research has increasingly attributed the immunomodulatory effects of Fuzi to Fuzi’s polysaccharides. At a dosage of 200 mg/kg, HSP polysaccharide notably elevated the serum levels of NO and IFN-γ in cyclophosphamide-induced immunocompromised mice. IFN-γ, a Th1 cytokine, can activate macrophages, while NO is produced by macrophages to eliminate pathogens (Fu et al., 2018). In another study, water-soluble polysaccharides derived from Fuzi showed significant antioxidant and immunomodulatory activities and promoted macrophage phagocytosis, which increased the secretion of macrophage-derived biofactors in RAW 264.7 cells (Yang et al., 2020). In a recent study, 200 mg mL^−1^ Fuzi neutral polysaccharide attenuated the suppression of immune organs and immune cells caused by cyclophosphamide (CTX) treatment by activating immune cells and promoting inflammation (Hu et al., 2023). In conclusion, Fuzi’s polysaccharides have shown potential in modulating immune responses, enhancing macrophage activity, and promoting cytokine production, thereby supporting its use in immunocompromised cases.

### 5.2 Pharmacokinetics

Unlike single-compound drugs, herbal extracts such as Aconiti Lateralis Radix Praeparata (Fuzi) exhibit a complex composition, which complicates their clinical use, particularly given Fuzi’s narrow therapeutic window due to its inherent toxicity. Understanding the pharmacokinetics of Fuzi and its primary chemical compounds—encompassing absorption, distribution, metabolism, and excretion—enables optimization of dosage, timing, and administration routes to maximize clinical efficacy and minimize toxicity (Zhang et al., 2014). Fuzi’s key components are diterpene alkaloids such as AC, MC, and HC, which also include less toxic derivatives like benzoylaconitine (BAC), benzoylmesaconitine (BMC), and benzoylhypaconitine (BHC).

#### 5.2.1 Absorption

##### 5.2.1.1 Fuzi extracts

AC shows low bioavailability (4.72% ± 2.66%) in rats, to which Fuzi extract (0.118 mg/kg AC) was administered intragastrically; however, its absorption is rapid, with a T_max_ of 58.00 ± 21.68 min. Pharmacokinetic parameters do not significantly change with repeated administration, although absorption rates increase. These findings suggest that other components in Fuzi may influence AC’s pharmacokinetic profile after prolonged exposure (Tang et al., 2012). Furthermore, the low bioavailability of Fuzi’s alkaloids is attributed to efflux transporter proteins, such as P-glycoprotein (P-gp), multidrug resistance protein 2 (MRP2), and breast cancer resistance protein (BCRP). These proteins serve as barriers by ejecting xenobiotic substances from the bloodstream back into the intestinal lumen, thus mitigating Fuzi’s toxicity. Studies have demonstrated that efflux transporter proteins mediate the uptake and transport of Fuzi active ingredients with the rule that the greater the toxicity of the alkaloids, the more efflux occurs, namely, AC, MA, HA > BAC, BMA, BHA (Yang et al., 2014).

##### 5.2.1.2 Monomer compounds of Fuzi

Research by Zhang et al. on three representative alkaloids—AC, benzoylaconitine (BAC), and aconine (ACN)—demonstrates that while all are rapidly absorbed, BAC shows the fastest absorption with a T_max_ of 0.31 ± 0.17 h. The C_max_ of the three were 10.99, 3.99, and 4.29 ngmL−1, respectively, reflecting that AC could be better absorbed compared to BAC and ACN. Despite their rapid uptake, these alkaloids are poorly absorbed overall, with P-gp inhibiting the uptake of AC and BAC, whereas ACN is absorbed passively (Zhang et al., 2015). Additionally, an *in vitro* intestinal absorption study of MC found that the absorption rate of MC was associated with concentration differences across the intestinal wall, indicating passive transport as its primary absorption mechanism (Yun et al., 2018).

#### 5.2.2 Distribution

Alkaloids are the primary active ingredients in Fuzi; understanding their tissue distribution is vital for elucidating Fuzi’s pharmacokinetics.

Wang et al. used HPLC-MS to study the distribution of AC in rats, finding the highest concentrations in the liver and lungs, followed by the heart, kidneys, and spleen, with notable individual variability (Wang et al., 2005). Another study on mice showed that AC was predominantly distributed in the liver and kidney, followed by the heart, blood, and brain. However, the results show significant alterations in distribution patterns in Mdr 1a −/− mice, with increased AC levels in all organs, especially the brain (Wu J. J. et al., 2018). A study about the Aconitum alkaloids’ distribution in the cadaver of a patient with acute aconite poisoning revealed the highest levels in urine, followed by bile, stomach, heart, and blood. Therefore, urine, blood, and bile can be used as the preferred bioassay in case of aconite poisoning (Liu et al., 2009). Niistu et al. found that Aconitum alkaloids were higher in the liver and kidney and lower in the heart and brain in autopsies of three suicide cases involving aconite poisoning. Moreover, the Aconitum alkaloid content was higher in the blood than in brain tissues (Niitsu et al., 2013).

ADAs, among the active ingredients of Fuzi, are considered less toxic alkaloids. According to available studies, ADAs are widely distributed in the heart, liver, lung, and kidney after entering the human body but less prevalent in the brain (Tao et al., 2023).

#### 5.2.3 Metabolism

##### 5.2.3.1 Drug-metabolizing enzymes (DMEs)

Cytochrome P450 enzymes (CYPs) are found in all organs and tissues, except for skeletal muscles and mature erythrocytes, with the highest abundance in the liver, gastrointestinal tract, lung, and kidney. These enzymes are crucial in metabolizing endogenous substances and exogenous compounds, including drugs and environmental chemicals. Notably, at least nine human hepatic cytochrome P450 enzymes are integral to drug metabolism, primarily through hydroxylation or oxidation processes that enhance solubility and facilitate excretion.

Tang et al. examined the cytochrome P450 isozyme changes during AC metabolism using human liver microsomes (HLMs) and recombinant CYPs, identifying six CYP-mediated metabolites in HLMs and eight recombinant CYP isoforms. Additionally, they found significant inhibition of AC metabolism by CYP 3A inhibitors, with moderate inhibition by CYP 2C9, 2C8, and 2D6, whereas CYP 2C19, 1A2, and 2E1 showed negligible effects. Aconitine is mainly transformed by CYP 3A4/5 and 2D6 into various metabolites, indicating the importance of these isoforms in its biotransformation (Tang et al., 2011; Jin et al., 2022). Since AC, MA, and HA have similar structures, studies have shown that the metabolic mechanisms of MA and HA are similar to those of AC. Ye et al. demonstrated that MA generates at least nine metabolites in the presence of HLMs. The main metabolic pathways include demethylation, dehydrogenation, hydroxylation, and dehydrodemethylation (Ye et al., 2010). Conversely, HA generates at least 11 metabolites, metabolized by demethylation, dehydrogenation, hydroxylation, dehydrodemethylation, and desdimethylation (Ye et al., 2011). In addition to DDAs, CYPs are involved in the metabolic reactions of MDAs. A total of 7, 8, and 9 metabolites of BAC, BMA, and BHA were found, respectively, in HLMs (Ye et al., 2013a).

Zhang et al. also investigated the inhibitory effects of aconitine on different isoforms of the CYP450 enzyme in rat liver microsomes *in vitro*. They concluded that aconitine inhibited the activities of CYP2C9 and CYP2D6 to a certain extent. Given that CYP2C9 and CYP2D6 are involved in the metabolism of commonly used clinical drugs, attention should be paid to the occurrence of drug metabolic interactions in the clinical use of Chinese medicines rich in aconitine alkaloids (Zhang, 2016).

##### 5.2.3.2 Efflux transporters

Efflux transporters (ETs), containing ATP-binding cassette proteins, mainly include P-gp, MRP2, and BCRP. These transporters are widely expressed in intestinal, hepatic, renal, and cerebral tissues and are involved in drug absorption, distribution, and excretion.

The role of efflux transporter proteins in alkaloid transport has received the attention of many researchers. Ye et al. performed bidirectional translocation assays of Aconitum alkaloids in the presence or absence of P-gp, BCRP, and MRP 2 inhibitors (cyclosporin A and verapamil, Ko 143, MK 571). Ultimately, they concluded that P-gp and BCRP are involved in the transport of AC, MA, and HA, while MRP2 may be implicated in the transport of several forms of these alkaloids (Ye et al., 2013b). While ETs are involved in Aconitum alkaloid transport, Aconitum alkaloids simultaneously affect ETs. AC increased the expression of P-gp, MRP 2, and BCRP in mouse jejunum, ileum, and colon. Diterpene alkaloids such as AC and benzoylglycyrrhetinic acid significantly increased MRP 2 and BCRP expression through activation of Nrf 2-mediated signaling pathways (Wu X. et al., 2018).

#### 5.2.4 Excretion

The kidney is the most important organ involved in the excretion of Aconitum alkaloids. In a case involving a 45-year-old male who ingested approximately 11 mg of DDAs, AC, MA, HA, and their hydrolyzed products were detected in the serum on the first day, with some alkaloids detectable in the urine even 6 days after intoxication. This suggests that Aconitum alkaloids undergo metabolism *in vivo*, and their hydrolysis products are excreted time-dependently in the urine (Mizugaki et al., 1998). Additionally, in a 40-year-old woman who died of aconite poisoning, higher levels of alkaloids were detectable in the kidneys, liver, and bile compared to other organs or serum, suggesting primary elimination of these alkaloids by the liver and kidneys. Meanwhile, the concentration of astragalus alkaloids in the ileum was also notably high., demonstrating that Aconitum alkaloids are eliminated through urine and feces (Ito et al., 2000).

## 6 Conclusion

Fuzi is a widely utilized herbal medicine in TCM. Recent studies have highlighted its potent pharmacological benefits coupled with significant toxicity. Processing Fuzi is recognized as an effective way to enhance its therapeutic effects while reducing its toxicity. This review addresses this topic through a wide range of Chinese and English research articles. Although Fuzi contains diverse chemical components, alkaloids, particularly DDAs, are its most active and toxic compounds. Improper clinical use of Fuzi can cause severe cardiotoxicity, hepatotoxicity, nephrotoxicity, and neurotoxicity, potentially leading to patient mortality. Consequently, traditional clinical practices have developed numerous processing methods to further influence the toxicity and pharmacological effects of Fuzi by altering the composition of its chemical components. Among these, salt processing particularly improves the pharmacological effects of Fuzi on the kidney (mainly including nephroprotective, anti-inflammatory, and immunomodulatory effects). However, research on processed Fuzi remains insufficient; further studies are urgently needed to explore its medicinal potential, particularly to understand its mechanism of action after processing.

## 7 Discussion

This review overviews the primary compounds, toxicity characteristics, kidney-related pharmacological effects, and processing methods of Fuzi. It outlines various processing techniques and introduces discussions on the mechanisms through which these methods enhance therapeutic effects and minimize toxicity. Notably, salt processing stands out for its unique ability to amplify Fuzi’s benefits for kidney health. However, this review does not exhaustively detail the changes in Fuzi’s chemical composition, toxicity, and pharmacological effects following processing, nor does it deeply explore the potential mechanisms by which different processing methods influence Fuzi. Current research on the chemical composition, toxicity, and pharmacological effects of processed Fuzi remains inadequate; the mechanisms underlying specific processing methods, such as salt processing, are poorly understood. Therefore, further investigations are essential to evaluate the impact of these processing techniques on the physicochemical properties of Fuzi and to elucidate their mechanisms. Additionally, future research should focus on clinical studies evaluating the safety and efficacy of Fuzi preparations for treating kidney disease. Through these studies, we can determine how far processing methods can extend the boundaries of Fuzi’s clinical applications, thereby supporting the identification of new methods and standards for Fuzi processing**.** In brief, the role and mechanisms of processing in improving the clinical efficacy and reducing the adverse effects of Fuzi and other Chinese herbal medicines hold significant potential for future research.
